# The Sit-and-Wait Hypothesis in Bacterial Pathogens: A Theoretical Study of Durability and Virulence

**DOI:** 10.3389/fmicb.2017.02167

**Published:** 2017-11-03

**Authors:** Liang Wang, Zhanzhong Liu, Shiyun Dai, Jiawei Yan, Michael J. Wise

**Affiliations:** ^1^School of Medical Informatics, Xuzhou Medical University, Xuzhou, China; ^2^Department of Clinical Pharmacology, Xuzhou Infectious Diseases Hospital, Xuzhou, China; ^3^School of Anaesthesia, Xuzhou Medical University, Xuzhou, China; ^4^Clinical Laboratory of Tuberculosis, Xuzhou Infectious Diseases Hospital, Xuzhou, China; ^5^School of Computer Science and Software Engineering, University of Western Australia, Perth, WA, Australia; ^6^The Marshall Centre for Infectious Diseases Research and Training, University of Western Australia, Perth, WA, Australia

**Keywords:** sit-and-wait hypothesis, pathogen, durability, virulence, energy storage mechanism, abiotic stress resistance

## Abstract

The intriguing sit-and-wait hypothesis predicts that bacterial durability in the external environment is positively correlated with their virulence. Since its first proposal in 1987, the hypothesis has been spurring debates in terms of its validity in the field of bacterial virulence. As a special case of the vector-borne transmission versus virulence tradeoff, where vector is now replaced by environmental longevity, there are only sporadic studies over the last three decades showing that environmental durability is possibly linked with virulence. However, no systematic study of these works is currently available and epidemiological analysis has not been updated for the sit-and-wait hypothesis since the publication of Walther and Ewald’s (2004) review. In this article, we put experimental evidence, epidemiological data and theoretical analysis together to support the sit-and-wait hypothesis. According to the epidemiological data in terms of gain and loss of virulence (+/-) and durability (+/-) phenotypes, we classify bacteria into four groups, which are: sit-and-wait pathogens (++), vector-borne pathogens (+-), obligate-intracellular bacteria (--), and free-living bacteria (-+). After that, we dive into the abundant bacterial proteomic data with the assistance of bioinformatics techniques in order to investigate the two factors at molecular level thanks to the fast development of high-throughput sequencing technology. Sequences of durability-related genes sourced from Gene Ontology and UniProt databases and virulence factors collected from Virulence Factor Database are used to search 20 corresponding bacterial proteomes in batch mode for homologous sequences via the HMMER software package. Statistical analysis only identified a modest, and not statistically significant correlation between mortality and survival time for eight non-vector-borne bacteria with sit-and-wait potentials. Meanwhile, through between-group comparisons, bacteria with higher host-mortality are significantly more durable in the external environment. The results of bioinformatics analysis correspond well with epidemiological data, that is, non-vector-borne pathogens with sit-and-wait potentials have higher number of virulence and durability genes compared with other bacterial groups. However, the conclusions are constrained by the relatively small bacterial sample size and non-standardized experimental data.

## Introduction

According to the conventional theories of evolution of virulence, all pathogens will end up in a relationship of commensalism or mutualism with their hosts, that is, being avirulent to their hosts (Ewald, 1983, 1987b). However, with more experimental data collected and theoretical studies conducted, the conventional wisdom underwent critical challenges and was overturned. Anderson and May are among the pioneers to use mathematics to model population dynamics of infectious diseases and proposed basic reproductive ratio *R*_o_ to measure pathogen fitness (Anderson and May, 1979, 1980, 1982; May and Anderson, 1979; Bull and Lauring, 2014), which is written as:

$$fitness∼R_{0}=\frac{transmission\,\;coefficient\left(\beta \right)*susceptible\,\;host\,\;density\,\;\left(S\right)}{natural\,\;mortality\,\;rate\,(\mu )*mortality\,\;caused\,\;by\,\;the\,\;{disease}^{\#}(\alpha )*\;re\,\mathrm{cov}er\,\;rate\left(\gamma \right)}$$

^#^Mortality caused by the disease is defined as bacterial virulence for its simplicity (Thomas and Elkinton, 2004).

Only when *R*_o_ > 1, that is, change rate $\frac{dY}{dt}$ in a population Y is larger than 0 where Y is the number of the infected, can virulence (disease) be maintained in the population (Anderson and May, 1979). Fitness, defined by *R*_o_, proposes that virulence can be achieved at a certain level with the influences of β, α, and γ individually, or interactively, and an optimal level of virulence can be reached when all parameters are appropriately balanced (Alizon et al., 2009). However, most models assume that parameters are coupled and a tradeoff exists, such as between recovery and virulence (Anderson and May, 1982), transmission and recovery (Alizon, 2008), and transmission and virulence (Ewald, 1983), etc.

Evolutionary biologist Paul W. Ewald was among the firsts to argue that bacterial transmission rate β and virulence α are linked (Ewald, 1983). In his work, costs and benefits tradeoff between transmission and virulence were systematically studied through epidemiological analysis, which validated the idea that non-vector-borne pathogens relying on direct host transmission are generally benign while those with vector-borne transmission mode are much more virulent (Ewald, 1983). From evolutionary perspective, a pathogen with direct transmission mode prefers maintaining a relatively low virulence phenotype because high virulence will lead to immobilization or death of the infected hosts, which incurs high costs by reducing contact opportunities with susceptible hosts. Without a vector, the pathogens would be trapped in dead or immobilized hosts and die out. Thus, even if a recent association between a pathogen and a host would lead to high virulence, the severity will over time reduce if the pathogen relies on host’s mobility for transmission (Ewald, 1983). On the other hand, if a pathogen were vector-borne, it would be risk-free to exploit hosts extensively without the impediment of host immobilization. Vectors such as mosquitoes, ticks, or flies can transfer pathogens from infected or immobilized hosts to a group of new susceptible population. In addition, since vector-borne pathogens rely on vectors for transmission, they tend to have lower virulence to their vectors than to their main hosts, which reinforces the virulence-transmission tradeoff theory (Elliot et al., 2003). A variety of pathogens that cause severe epidemics to human society fall into this category, such as *Yersinia pestis* (vector: fleas; disease: Bubonic Plague), *Borrelia burgdorferi* (vector: ticks; disease: Lyme disease), and *Francisella tularensis* (vector: ticks and flies; disease: Tularemia), etc. (Perry and Fetherston, 1997; Gubler, 2009; Conlan, 2011; Cook, 2015).

During a study of transmission modes of pathogens and their virulence, a set of non-vector-borne pathogens was identified as highly or extremely highly virulent bacteria, such as *Mycobacterium tuberculosis* and *Corynebacterium diphtheriae*, etc. (Ewald, 1983; Walther and Ewald, 2004). Careful study of these outliers showed that they are actually a special instance of vector-borne pathogens that have long-lived free-living stages during their infection cycles. The reasoning is as simple as that long-lived pathogens in the external environment can live long enough to wait for potential hosts to reach them. Thus, they are able to be highly virulent in their hosts without incurring high costs, just like the vector-borne pathogens (Ewald, 1987a). In fact, high virulence and long environmental longevity were initially studied in a Susceptible-Infected-Recovery (SIR) model by Anderson and May because they cause cyclic changes of host abundance (Anderson and May, 1980). However, interactions between the two parameters were not further explored. Ewald studied those models (Ewald, 1987a) and originally proposed that pathogenic bacteria with high durability in the external environment tend to evolve toward higher virulence phenotypes because they do not rely on the mobility of their hosts for transmission and will have lower costs and higher gains by incurring the immobility of their hosts (Ewald, 1983, 1987a, 2004; Walther and Ewald, 2004). Those bacteria are therefore termed sit-and-wait pathogens (Ewald, 1987a).

**FIGURE 1:**
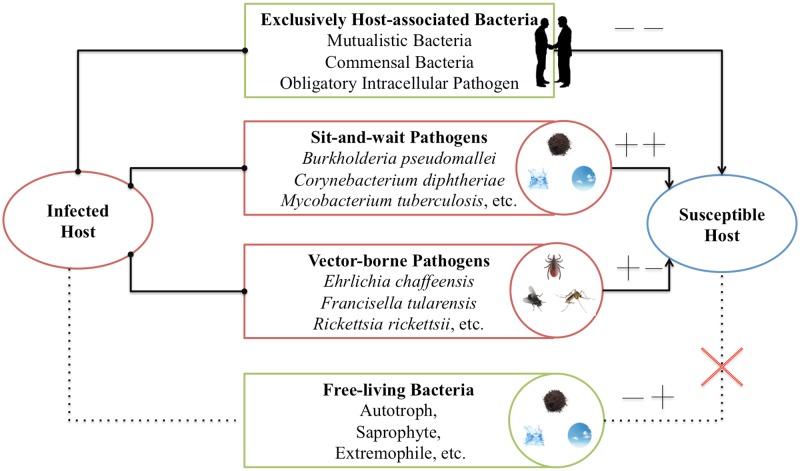
Classification of bacteria based on gain (+) or loss (+) of durability and virulence phenotypes. In this classification, four groups are included: exclusively host-associated bacteria (-), sit-and-wait pathogens (++), vector-borne pathogens (+-), and free-living bacteria (-+).

Walther and Ewald (2004) collected data about the survival time and mortality-rate of a set of human respiratory pathogens to support the sit-and-wait hypothesis. The reason to limit the study to human respiratory pathogens was to eliminate variation due to host species or site of infection (Walther and Ewald, 2004). However, considering that sit-and-wait hypothesis should be a general mechanism for bacterial virulence evolution, there is no need to restrict the investigation to respiratory pathogens only. In this study, based on the gain and loss of virulence (+/-) and durability (+/-) phenotypes, we divided bacteria into four categories (**Figure 1**): exclusively host-associated bacteria (-/-), sit-and-wait pathogens (+/+), vector-borne pathogens (+/-), and free-living bacteria (-/+). For each category, epidemiological data are collected for bacterial mortality and survival time based on literature mining in order to investigate the relationship between durability and virulence, that is, whether higher durability leads to higher virulence.

A recent experimental study found that environmental durability can diversify the population structure and virulence strategies of a fish pathogen, that is, longer survival time corresponds to higher virulence (Sundberg et al., 2014). For sit-and-wait pathogens, in order to survive hash external environment and maintain viability, they have to deal with a vast range of abiotic stresses, such as temperature fluctuation, hyperosmolarity, pH change, and hazardous radiation, etc. Thus, their survival depends on their ability to sense and respond to the ever changing environment (Boor, 2006). In addition, maintenance of viability requires careful consumption of large amount of energy storage compounds, otherwise known as ‘energy of maintenance’ (Wilkinson, 1963; Roszak and Colwell, 1987). Thus, energy reserve is another important factor for bacterial survival, especially when considering that bacteria need to adapt to extensive changes after shedding from hosts to the external environment where they are deprived of nutrients. In brief, bacterial energy storage mechanisms (ESM) and abiotic stress resistance (ASR) are two major factors for bacterial survival in the external environment. Why this is the case is discussed below.

Bacteria consume energy all the time for their regular activities, ranging from mobility to reproduction. Thus, energy storage compounds are an indispensable element for bacterial durability due to the fluctuation of nutrients in the external environment. Some bacteria have one or more types of energy reserves and can therefore be very durable in the external environment, e.g., *Corynebacterium diphtheriae*, that accumulates glycogen, and *Mycobacterium tuberculosis* (Mtb), that accumulates both wax ester and glycogen, while other obligate intracellular bacteria such as *Ricketssia* and *Borrelia* do not accumulate any energy reserves and can only survive inside hosts (Wang and Wise, 2011). Coincidentally, the former two bacteria are more highly virulent pathogens than the latter two obligate intracellular pathogens. In fact, several studies have already linked one of the major energy reserves, glycogen, with bacterial persistence, colonization, and virulence (Wilson et al., 2010). Henrissat et al. (2002) screened 55 completely sequenced bacterial genomes and found that most of the free-living bacteria accumulate glycogen as an energy reserve while those with parasitic lifestyles do not, and concluded that glycogen accumulation can be a marker for bacterial parasitic behavior. Thus, bacterial energy reserves are very much likely to be an indicator for bacterial durability, lifestyle, and probably virulence. Currently, there are five major storage compounds identified in bacteria: triacylglycerol (TAG), wax esters (WE), polyhydroxybutyrate (PHB), polyphosphate (PolyP), and glycogen (Wang and Wise, 2011), all of which will be investigated in this study.

Bacteria exist almost everywhere on the planet from deep sea (*Cowellia hadaliensis*) to nuclear reactors (*Deinococcus radiodurans*) to inside the human body (*Escherichia coli*) (Kato and Qureshi, 1999; Marles-Wright and Lewis, 2007). Accordingly, bacteria are experts in evolving sufficient mechanisms to deal with and/or adapt to harsh and fluctuating environments. Although many proteins have been reported to protect bacteria from harsh conditions, they are normally synthesized only when bacteria sense the corresponding stresses, which can save bacterial energy and improve working efficiency. Stress sensing is generally the first step for bacteria to react to abiotic stresses. Specifically, when sensing a stress, transcription factors, such as sigma factors, etc., will be activated to interact with RNA polymerase and expression of stress responsive genes will be unregulated. These genes encode stress proteins that are involved in dealing with stresses directly (Marles-Wright and Lewis, 2007). Thus, the former are termed general stress genes while the later are termed as specific stress genes (Schumann, 2007). Currently, there are three types of sensors: DNA, mRNA, and proteins, among which, DNA and mRNA may sense stresses by changing their conformations, which change gene expression level by influencing transcription-related protein binding (Schumann, 2007). However, these sensors cannot be detected by screening proteomes. In addition, only protein sensors are counted as major stress sensors, which include molecular chaperones, proteases, kinases, and sigma factors, etc. (Schumann, 2007). Since these protein sensors are rather diverse, forming sophisticated networks, and remote from the actual stresses, we will not review them specifically. However, a set of proteins that are more proximal to the actual stresses have been collected and used for studying the sit-and-wait pathogens, that is, proteins protecting bacteria from reactive oxygen species (ROS), UV radiation, and desiccation, etc. In addition to the three typical abiotic stresses introduced above, there are still many other abiotic stresses in the external environment, such as high salinity and extreme temperature, etc. Obviously, resistance to abiotic stress can increase the opportunities for pathogens to survive longer in unfriendly environments. For a complete list of ASR types, please refer to Supplementary Table S1.

In this study, we constructed two sets of hidden Markov models (HMMs) in terms of ESM and ASR. For each bacterial category, five representative proteomes were screened using the HMM models for distribution of ESM- and ASR-related genes. In addition, virulence factors collected from VFDB were also searched in the proteomes in order to identify homologous sequences (Chen et al., 2016). It was hypothesized that the distribution patterns of durability and virulence genes should be consistent with epidemiological data and also with the four-way classification of bacteria described above. The proposal of sit-and-wait hypothesis had spurred a debate about its validity both theoretically (Bonhoeffer et al., 1996; Walther and Ewald, 2004; Alizon et al., 2009; Roche et al., 2011) and experimentally (Tolba et al., 2007; Ayyadurai et al., 2008; Sundberg et al., 2014). While Walther and Ewald (2004) collected abundant epidemiological data on respiratory pathogens supporting the existence of a positively correlation between durability and virulence in 2004, no systematic study of this hypothesis has been conducted since then. Only sporadic scientific articles are available so far to support the hypothesis (Tolba et al., 2007; Jani and Cotter, 2010; Vezzulli et al., 2010; Sundberg et al., 2014). In this study, we searched the literature linked with sit-and-wait hypothesis tracing back through several decades. We also put experimental evidence and epidemiological data together in order to examine the validity of this intriguing hypothesis. In addition, we endeavored to correlate bacterial durability with virulence using bioinformatics methods so as to facilitate the investigation and provide a preliminary guide to test the hypothesis.

## Materials and Methods

### Collection of Bacterial Proteomes and Epidemiological Data

For the sit-and-wait pathogens, proteomes of eight representative bacteria were selected. For the other three categories, only proteomes of five representative bacteria were sourced. All proteomes are downloaded from UniProt (The UniProt Consortium, 2017). In order to make the investigation more persuasive, we included quantitative data, such as external survival time and host mortality, for comparison. For details, please refer to **Table 1**. All eight sit-and-wait bacteria were selected based on a literature search using key words such as “sit-and-wait,” “pathogenic,” and “bacteria,” in PubMed and Google Scholar databases. Representative bacteria from other categories were also selected using the same method. All absent data were further sourced from government databases, medical and biological textbooks, and also referenced papers. The collected epidemiological data are only representative and should not be considered to be comprehensive due to the large volume of literature available. It only serves to provide preliminary support for the linkage between durability and virulence.

**Table 1:** Epidemiological analysis of 8 non-vector-borne environmental human pathogens with sit-and-wait potentials.

Bacterial pathogen	Disease	Survival (days)	Mortality (M/I)%	Reference
*Acinetobacter baumannii*	Pneumonia	27	10.6%	Jawad et al., 1998; Sunenshine et al., 2007
*Burkholderia pseudomallei*	Melioidosis	>120	16.5%	Shams et al., 2007; Chien et al., 2015; Kamjumphol et al., 2015
*Corynebacterium diphtheriae*	Diphtheria	180	36.5%	Kramer et al., 2006; Dandinarasaiah et al., 2013
*Haemophilus influenzae*	Bacteremia	12	10–30%	Peltola, 2000; Kramer et al., 2006
*Leptospira interrogans*	Leptospirosis	>42	4–13%	Hellstrom and Marshall, 1978; Costa et al., 2015
*Mycobacterium tuberculosis*	Tuberculosis	>360	12.3%	Ghodbane et al., 2014; Lin et al., 2014
*Pseudomonas aeruginosa*	Pneumonia	18	18–61%	Kang et al., 2003; Khaniki et al., 2014
*Yersinia pestis*	Pneumonic plague	280	100%	Ayyadurai et al., 2008; Pechous et al., 2016


*Survival means maximal survival time sourced from corresponding literature. Mortality means maximal death rate sourced from corresponding literature.*

### Construction of HMMs Related to Energy Storage Mechanisms

Enzymes directly linked with energy storage metabolisms in bacteria were first collected through a comprehensive up-to-date review of literature (Lopez et al., 1998; Henrissat et al., 2002; Zhang et al., 2002; Ishige et al., 2003; Kalscheuer and Steinbuchel, 2003; Brown and Kornberg, 2004, 2008; Kulaev et al., 2004; Uchino et al., 2007; Rao et al., 2009; Finkelstein et al., 2010; Kalscheuer, 2010; Achbergerova and Nahalka, 2011; Wang and Wise, 2011; Whitehead et al., 2014; Albi and Serrano, 2016). For a complete list of all the enzymes and their distributions in bacterial proteomes, please refer to Supplementary Table S1 of the review by Wang and Wise (2011). All sequences of ESM-related enzymes were downloaded from The UniProt Consortium (2017). HMMs of all enzymes were constructed by using HMMER based on multiple sequence alignments downloaded directly from Pfam database (Eddy, 2003; Finn et al., 2010). The command, hmmsearch, was used to search all proteomes for homologous sequences of ESM-related enzymes with E-value set to 1e-50. Complete gain or loss of all enzymes in the metabolism of an energy storage compound was marked as 1 and 0, respectively. Short Python scripts (Cock et al., 2009) were written and executed during the processes of file handling and text mining in order to integrate data from hmmsearch results into Supplementary Table S2.

### Construction of HMMs Related to Abiotic Stress Resistance

Seed sequences of ASR related proteins were selected by using a web-based browser for Gene Ontology (GO) terms and annotations, QuickGo (provided by the UniProt-GOA group at EBI) (Binns et al., 2009). Database was filtered by designating the taxonomic group as bacteria (Taxonomy Identifier = 2). Meanwhile, 35 GO identifiers under the category of response to abiotic stimulus (GO:0009628) were selected for filtering meaningful and important proteins associated with ASR. A complete list of the 35 GO identifiers is presented below in Supplementary Table S1. In total, 1406 bacterial proteins were collected as seed proteins and used for constructing HMMs corresponding to ASR after using Perl script nrdb90.pl to remove the sequences with more than 90% similarity from the selected proteins (Holm and Sander, 1998). After obtaining sequences for all seed proteins, remote BLAST was performed to collect homologous sequences for each seed protein from the NCBI non-redundant database of protein sequences. In addition, since the sequences returned from remote BLAST were in extensible markup language (XML) file format, a Python script for parsing XML file was also written to extract the homologous sequences. The standalone command-line version of MUSCLE was used so the MSAs were done automatically (Edgar, 2004). Heads or tails of multiple sequence alignments tend to be more inconsistent (Wise, 2010). Thus all MSAs were manually edited to remove heads and tails by using JalView, a free program for MSA editing, visualization, and analysis (Waterhouse et al., 2009). HMMER was selected for the construction of HMMs through hmmbuild command by using multiple sequence alignments. Since HMMER only recognizes STOCKHOLM format, all MSAs results were converted from FASTA to STOCKHOLM format. For detailed steps and python scripts of HMM construction from scratch, please refer to Wang (2013). Python scripts and HMM model for bacterial ASR and ESM are both available under request. For searching homologs, routine procedures are performed by following HMMER User’s Guide (Eddy, 2003). In order to avoid the influence of bacterial proteome size, the number of ASR genes was converted into the percentage of ASR genes in bacterial proteome.

### Homologs of Bacterial Virulence Factors

VF sequence package includes 2595 non-redundant core bacterial virulence factors sourced from VFDB (downloadable from VFDB website http://www.mgc.ac.cn/VFs/) (Chen et al., 2005). BLAST-equivalent phmmer command from HMMER package was recruited to automatically search VF homologs in all selected proteomes by using E-value of 1e-50. For usage of HMMER package please refer to the protocol document (Eddy, 2003). Results were all under further processed through a set of short python scripts (Cock et al., 2009). In order to avoid the influence of bacterial proteome size, number of VFs was converted into percentages of VFs in the respective bacterial proteomes.

### Statistical Analysis

Unless otherwise indicated, Student’s *t*-test was used for all statistical analyses via a Python script, specifically the scipy statistical computation package. Data used in the comparisons can be found in the Tables. Where a range is given, the midpoint in the range has been used in the statistical analyses.

## Results and Discussion

**FIGURE 2:**
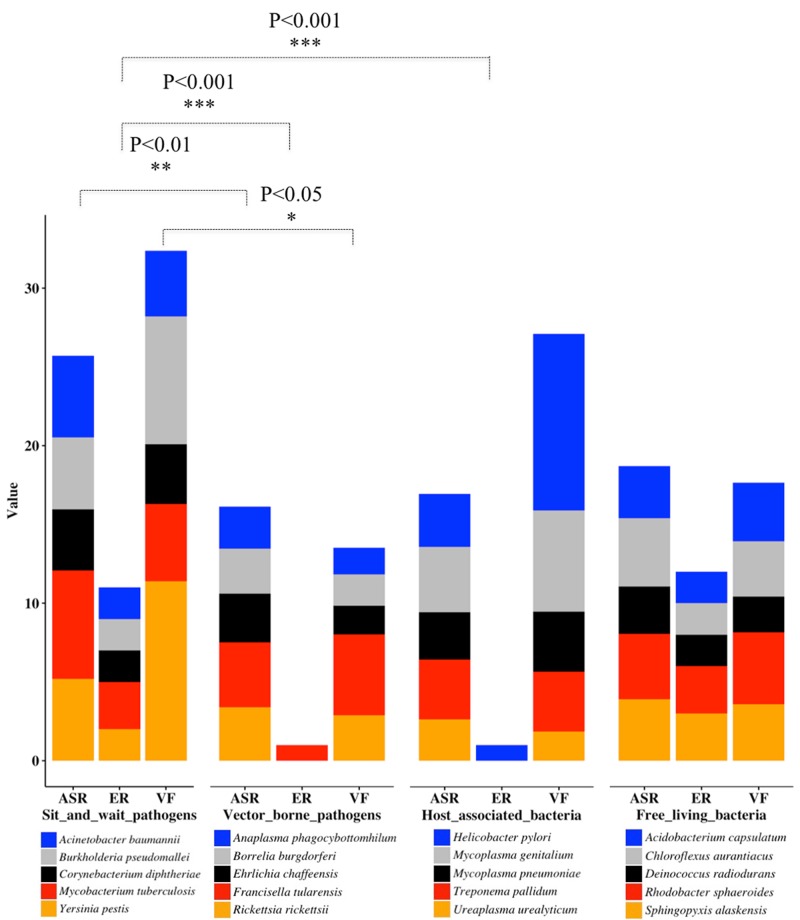
Stacked column chart for the illustration of bacterial classification based on distribution patterns of durability and virulence genes as previously defined. For each category, only five representative bacterial species are displayed. Energy Reserve (ER) includes glucose, polyphosphate, triacylglycerol, wax ester, and polyhydroxybutyrates. Value of ER represents total number of energy storage compound types. Value of ASR represents added percentages of genes related to starvation, temperature, osmolarity, pH, and desiccation, etc. Value of VF means added percentages of bacterial virulence factors. For statistical analysis, we classify the results into three levels, ^∗∗∗^*P* < 0.001, ^∗∗^*P* < 0.01, and ^∗^*P* < 0.05.

Through literature review and data mining, we collected bacterial durability and mortality data for representative bacteria in the previously defined four groups, which are: exclusively host-associated bacteria, sit-and-wait pathogens, vector-borne pathogens, and free-living bacteria. The results are summarized in **Table 1** for sit-and-wait pathogens and in **Table 2** for other categories, respectively. Correlation between durability and virulence phenotypes were calculated within and between groups. After that, HMMs were used to search for distribution patterns of enzymes related with energy storage metabolism and ASR. Homologous sequences of core virulence factors in each bacterium were also searched. The combined result is presented in **Figure 2** with details summarized in Supplementary Table S2. Relationships between durability and virulence based on bioinformatics analysis are presented exclusively for each bacterial group. In addition, bacterial proteome size was also identified as an important factor for bacterial grouping and lifestyle based on our analysis.

**Table 2:** Bacterial classifications in terms of environmental survival and mortality.

Obligate intracellular	Disease	External survival (hours)	Mortality^##^ (M/I)%	Reference
*Helicobacter pylori*	Gastric ulcer	< = 1.5	–	Kramer et al., 2006
*Mycoplasma pneumoniae*	Mild pneumonia	4	–	Wright et al., 1968; Kashyap and Sarkar, 2010
*Mycoplasma genitalium*	Urethritis	1	–	Waites, 2003
*Treponema pallidum*	Syphilis	–	–	van der Sluis et al., 1985
*Ureaplasma urealyticum*	Urethritis	1	–	Poulin et al., 1979
**Vector-borne**	**Disease**	**External survival (days)**	**Mortality (M/I)%**	**Reference**
*Anaplasma phagocytophilum*	Anaplasmosis	<18	0.6%	Kalantarpour et al., 2000; Dahlgren et al., 2011
*Borrelia burgdorferi*	Lyme disease	<45	<0.03%	Badon et al., 1989; Kugeler et al., 2011
*Ehrlichia chaffeensis*	Ehrlichiosis	11	3%	McKechnie et al., 2000; Zhang et al., 2007
*Francisella tularensis*	Tularemia	60	>30%	Mironchuk and Mazepa, 2002; Titball and Sjostedt, 2003
*Rickettsia rickettsii*	Typhus	–	2–30%	Ryan and Ray, 2004; Azad, 2007
**Free-living**	**Disease**	**External survival (days)**	**Mortality (M/I)%**	**Reference**
*Acidobacterium capsulatum*	–	+∞	–	Kishimoto et al., 1991
*Chloroflexus aurantiacus*	–	+∞	–	Tang et al., 2011
*Deinococcus radiodurans*	–	+∞	–	Makarova et al., 2001
*Rhodobacter sphaeroides*	–	+∞	–	Kontur et al., 2012
*Sphingopyxis alaskensis*	–	+∞	–	Ting et al., 2010


*^##^Indicates excluding prenatal mortality. - represents data not available.*

### Statistical Analysis of Host Mortality versus Species Durability

Eight representative non-vector-borne human pathogens were collected based on literature review and first studied in terms of external survival time and host mortality. All these pathogens have common features, that is, long environmental survival time and high mortality (**Table 1**) when compared with bacteria in the other three categories (**Table 2**). According to the sit-and-wait hypothesis, higher durability should facilitate higher virulence. However, linear regression of host mortality (percentage) against persistence in the environment (in days) across the data in **Table 1** only yielded a modest, and not statistically significant, correlation (*r*-value 0.387, *P*-value 0.344). While this may be due to the small sample size and the use of statistics from non-standardized experiments for the survival and mortality measurements, it is also understandable given the argument that durability is an enabler of virulence, but is not causally linked.

In order to better understand the selected bacteria and their sit-and-wait potentials, we dive into each species for a detailed investigation. *Acinetobacter baumannii* is a ubiquitous Gram-negative bacterial pathogen in the environment, which is capable of causing a wide range of infections and ranked among the top 10 pathogens causing septicemia (Jawad et al., 1998). Studies have shown that *A. baumannii* can persist in hospital environments for up to 3 years (Sherertz and Sullivan, 1985). However, a systematic study found out that multi-drug resistant (MDR) and susceptible strains of *A. baumannii* have similar survival times in distilled water, for a mean time of 27 days (Jawad et al., 1998). As for mortality, MDR *A. baumannii* shows a higher rate (26%) than the susceptible strain (17.6%) (Sunenshine et al., 2007), which may be caused by more virulence factors in the MDR strains rather than survival time in the environment. Since the mortality rate of uninfected control is 11.2% (Sunenshine et al., 2007), the average mortality rate for *A. baumannii* is re-adjusted to10.6%. *Burkholderia pseudomallei is* a Gram-negative bacillus and the causative agent of Melioidosis with varied severity and difficulty of treatment (Shams et al., 2007). It is normally dwelling on inmate surfaces and can survive in soil microcosms for at least 120 days (Shams et al., 2007; Kamjumphol et al., 2015). A recent study of a *B. pseudomallei* outbreak in Singapore between 2001 and 2010 hospital showed that the bacteria has a high mortality of 16.5% among 170 patients (Chien et al., 2015). Survival of *Corynebacterium diphtheriae* on dry surface is reported to be up to 6 months (Kramer et al., 2006). As a leading cause of childhood mortality in developing countries, a study of diphtheria spanning 12 years in a hospital found that the mortality rate is as high as 36.5% (Dandinarasaiah et al., 2013). *Haemophilus influenzae* is capable of surviving on a dry surface for 12 days and of causing a variety of clinical manifestations with a mortality rate worldwide ranging from 10 to 30% without vaccination (Peltola, 2000; Kramer et al., 2006).

*Leptospira interrogans* is a worldwide zoonotic pathogen and causes the emerging infectious disease Leptospirosis (Levett, 2001). Its survival in a typical New Zealand soil is at least 42 days (Hellstrom and Marshall, 1978). A systematic study showed that the worldwide mortality rate of Leptospirosis ranges from 4 to 13% between 1970 and 1999 (Costa et al., 2015). *Mycobacterium tuberculosis* is a typical sit-and-wait pathogen. It causes serious public health issues and its estimated overall mortality rate is 12.3%, based on case studies between 2003 and 2007 (Lin et al., 2014). Its viability and virulence in soil as *Mycobacterium tuberculosis* complex (MTC) lasts more than 12 months (Ghodbane et al., 2014). *Pseudomonas aeruginosa* is a major nosocomial pathogen with high morbidity and mortality that ranges from 18 to 61% (Kang et al., 2003) and can survive in bottled water for more than 50 days (Khaniki et al., 2014). However, the survival time heavily relies on temperature, with high temperature leading to better viability. Although *Yersinia pestis* has a vector-borne transmission route and causes endemic bubonic plague, it can also be transmitted from human-to-human via air droplets and causes pneumonic plague (Galimand et al., 2006). It has the ability to survive and remain virulent in the external environment for more than 280 days, which makes it one of most dangerous sit-and-wait bacterial pathogens (Ayyadurai et al., 2008). Without antibiotic treatment, the death rate would be 100% within 24-h infection, while mortality of treated patients can approach 50% (Pechous et al., 2016).

Vector-borne pathogens tend to have a comparatively shorter external survival time or no lifecycle out of hosts/vectors at all, due to their highly developed adaptation to their host environment. For example, *Rickettsia rickettsii* cannot survive outside hosts (Azad, 2007). However, our review of the literature did not found survival times of *Anaplasma phagocytophilum*, *Borrelia burgdorferi*, and *Ehrlichia chaffeensis* in the environment. The reported external survival time of the three pathogens in **Table 2** is actually their viability in *in vitro* cells or blood products under refrigeration conditions (Badon et al., 1989; Kalantarpour et al., 2000; McKechnie et al., 2000). Thus, these pathogenic bacteria could be very sensitive to environmental stresses and can only survive within host or vector cells. *F. tularensis* is an exception and is able to survive in the water system for up to 2 months (Mironchuk and Mazepa, 2002), which suggests that *F. tularensis* may have multiple transmission routes, hence, higher virulence. The vector-borne pathogens normally have high mortality rate (Titball and Sjostedt, 2003; Ryan and Ray, 2004). Although Lyme disease is reported to be rarely fatal (Kugeler et al., 2011), if left untreated, the mortality rate of the disease can be high. A similar situation also applies to *A. phagocytophilum* and *E. chaffeensis* infections. Obligate intracellular bacteria (commensal, symbiotic, and pathogenic) are all exclusively associated with hosts, with extremely weak environmental survival ability and do not contribute to host illness and mortality or only cause mild syndromes to the host. The maximal survival time falls into the scale of several hours. Finally, free-living bacteria generally have long survival times but do not have the ability to attack humans, so there is no reported mortality. For representative examples, please refer to **Table 2**.

Comparative analysis of the four bacterial groups in terms of external survival and mortality shows obvious differences among each other. Sit-and-wait pathogens generally have higher mortality rate and strong durability in the environment. Although vector-borne pathogens seem to be long-lived and highly pathogenic (**Table 2**), the survival data come from persisting in *in vitro* cells and blood samples. There are insufficient reports relating to their environmental survival. As for obligate intracellular bacteria, due to their high dependence on hosts, both the durability and mortality are inconsequential. Finally, free-living bacteria display effectively infinite survival times in the environment but no host mortality. The epidemiological data clearly separate the bacteria into four groups according to the durability and mortality factors. In addition, high durability and virulence are apparently associated with sit-and-wait pathogens.

### Bioinformatic and Statistical Analysis of Energy Reserves

When *t*-tests were used to compare the counts of durable-energy-reserve pathways in the sit-and-wait species versus the other groups of species, we noted that sit-and-wait species have significantly more than both host-associated (*P*-value 0.000105) and vector-born (*P*-value 0.000105) species, but the counts of energy reserve pathways in the sit-and-wait species are similar to those found in free-living species (*P*-value 0.545) (**Figure 2**). That is, sit-and-wait pathogens and free-living bacteria normally use more than one storage compound for energy metabolism, while vector-borne and intracellular bacteria seem to almost completely lose the ability to accumulate and utilize energy compounds. This expands the conclusion of Henrissat et al. (2002) that glycogen metabolism loss could be a marker for bacterial parasitic lifestyle.

Among the five energy storage reserves studies, wax ester (WE) is a neutral lipid that is accumulated as intracellular storage compounds by a few bacteria, such as *Acinetobacter* spp. and *Rhodococcus opacus* (Ishige et al., 2003). In addition, WE is also reported to be detected in the following bacteria: *Moraxella*, *Micrococcus*, *Fundibacter*, *Neisseria*, *Marinobacter*, *Pseudomonas*, *Corynebacterium*, *Mycobacterium*, and *Nocardia* (Ishige et al., 2003; Waltermann and Steinbuchel, 2005). A recent study shows that the final enzyme responsible for WE synthesis in bacteria is the bifunctional wax ester synthase/acyl-coenzyme A (acyl-CoA): diacylglycerol acyltransferase (WS/DGAT), which exhibits both acyl-CoA: fatty alcohol acyltransferase (WE synthesis) and acyl-CoA: diacylglycerol acyltransferase (TAG synthesis) activity (Kalscheuer and Steinbuchel, 2003). Thus, TAG and WE share the same final synthesis step. Both TAG and WE possess higher calorific value than carbohydrates and proteins, which means that when oxidized, more energy is released. That is why these reserves are able to support the environmental persistence of sit-and-wait bacteria. A recent study by Sirakova et al. (2012) shows that the dormancy of TAG and WE deficient Mtb strains via inactivating fatty acyl-CoA reductase genes is disrupted with increased cell wall permeability, high metabolic activity, and also impaired antibiotic tolerance (Sirakova et al., 2012), which indicates that the two energy storage compounds play essential roles in the environmental persistence of Mtb. Another bacterial energy reserve PHB acts as an osmotically neutral compound for carbon and energy storage (Uchino et al., 2007). Many bacteria accumulate it during stationery phase, especially when nitrogen is limited and a carbon source is abundant, which makes it a more widespread energy reserve than TAG and WE in bacteria. Sporadic studies have already proposed that accumulation of PHB can increase bacterial survival ability (Lopez et al., 1998). For example, *B. pseudomallei*, which does not store glycogen at all but is able to accumulate and utilize PHB, is rather durable in the external environment (Inglis and Sagripanti, 2006).

PolyP is a linear polymer of 100s of inorganic phosphate residues linked together by higher-energy phosphoanhydride bonds. It is ubiquitous in Nature and has been claimed to be present in all bacteria (Rashid et al., 2000a). In addition, it is labile and rapidly used up. Thus, there is no much literature studying its distribution in bacteria although it has been linked with bacterial mobility, biofilm development, long-term survival, and virulence (Rashid et al., 2000a,b; Kim et al., 2002). Last, but not the least, among the five energy reserves is glycogen, a homopolysaccharide consisting of glucosyl residues only. Currently, glycogen has been reported to exist in more than 50 bacteria across all bacterial species (Preiss, 2009). Theoretical study suggests that more than 300 bacterial proteomes harbor a complete glycogen metabolism pathway (Wang and Wise, 2011). According to numerous experimental studies, glycogen is strongly linked with bacterial lifestyle, persistence, and virulence, etc. (Strange, 1968; Bonafonte et al., 2000; Henrissat et al., 2002; Wilson et al., 2010).

Individually, each of the five energy storage compounds contributes to bacterial durability. With more than one ER in a bacterium, it would be strong advantage for the species to survive harsh conditions. This also explains why sit-and-wait pathogens normally harbor more than one type of energy reserves. However, only glycogen and PolyP metabolism have been shown experimentally to impact bacterial virulence at currently stage, which links durability and virulence together. As for WE, TAG, and PHB, more studies are required and similar tests should be performed in order to check their contributions to the virulence of pathogenic bacteria at molecular level.

### Bioinformatic and Statistical Analysis of Abiotic Stress Resistance

Analysis of the second factor, ASR, provided similar groupings. The group of sit-and-wait species has a significantly larger percentage of ASR proteins compared with the host-associated species (*P*-value 0.0143, versus threshold of 0.05) and compared with the vector-born species (*P*-value 0.00871). This is understandable, as host-associated species and vector-born species are largely shielded from the external environment. Interestingly, the sit-and-wait species also have a significantly higher percentage of ASR protein when compared with the free-living species (*P*-value 0.0357). We speculate that this may be because sit-and-wait pathogens have not only to deal with a hostile external environment, but also host immune system attack.

### Bioinformatic and Statistical Analysis of Virulence Factors

The situation for the third factor, presence of virulence factors, is a little more complicated. It can be argued that the number of virulence factors do not represent the degree of bacterial virulence due to the influences of gene transcription and post-translational modification. This is to some level true. However, the number of VFs is a sufficient indicator to reflect the diverse mechanisms that bacteria are armed with to cause harm in hosts, which is an important aspect of bacterial virulence. Analyzing the percentages of virulence factors in Supplementary Table S2 as a proxy for virulence, we found that sit-and-wait pathogens have a significantly higher percentage than vector-born species (*P*-value 0.0442), a higher percentage (but not significantly so) than free-living species and a similar percentage to host-associated species (*P*-value 0.639). The first of these results is likely due to vector-born pathogens needing to evade the vector immune systems of both the vector species and the eventual host, which may constrain the range of virulence factors. The vector-borne pathogen *F. tularensis* appears to be an outlier in this group with its large percentage of virulence factors, which matches its mortality very well. ESM analysis also shows that *F. tularensis* has the complete glycogen metabolism pathway. Although it is accepted as a vector-borne pathogen, it is also reported to have long-term survival ability, which makes it a potential pathogen similar to *Yersinia pestis* for multi-modal transmission (Mironchuk and Mazepa, 2002). Further investigation into the composition of the virulence factors should be performed for a better understanding of this phenomenon.

Comparison of sit-and-wait pathogens and host-associated bacteria in terms of the number of virulence factors did not find significant difference. Such a result is reasonable since host-associated bacteria also need to invade and colonize hosts, which should necessitate them being equipped with a set of VFs. As for non-pathogenic free-living bacteria, it is a little bit confusing to see that they have considerable number of virulence factors in the proteomes. The reason for the paradox is that virulence factors have also been identified in non-pathogenic bacteria (Niu et al., 2013). Keen reported that non-pathogenic bacteria are able to encode VFs like types III and IV secretion systems previously considered unique to pathogens (Keen, 2012). These findings make the classical definition of virulence factors controversial. A recent study classified VFs into two categories: pathogen-specific VFs and general VFs and general VFs are common in non-pathogenic bacteria (Niu et al., 2013). In our preliminary study, we used all VFs as a single group in order to search proteomes thoroughly for all VFs. For future work, we will make our screening subtler by using different categories of VFs, which might differentiate distribution patterns of VFs between bacterial groups.

### Proteome Size and Bacterial Lifestyles

Genome reduction is the dominant theory for bacteria transiting from free-living to facultative and finally to intracellular bacteria with parasitic or symbiotic lifestyles, and has been widely observed in Nature. Examples include *Mycoplasma* and *Rickettsia* (Moran, 2002; Wolf and Koonin, 2013). For the 20 bacteria listed in Supplementary Table S2 separated by transmission modes, the analysis of bacterial proteome sizes shows obvious differences between the four groups. The average proteome sizes for sit-and-wait, vector-borne, host-associated, and free-living bacteria are 3989, 1295, 855, and 3547, respectively. Statistical analysis shows that proteome sizes of sit-and-wait pathogens are significantly different from vector-borne (*P*-value 0.00351) and host-associated bacteria (*P*-value 0.00177), but are not significantly different from those of free-living bacteria (*P*-value 0.543). Considering that both sit-and-wait pathogens and free-living bacteria have a long-term external survival period, they need to adapt to different niches and cope with a variety of stresses. These results are consistent with epidemiological data in terms of bacterial lifestyle. Secondly, distribution patterns of ESMs (Wilkinson, 1963) shown in **Figure 2** match with transmission modes and also correlate with bacterial proteome sizes.

### Potential Targets for Testing the Linkages between Durability and Virulence

Theoretical analysis in this study shed light on the relationship between durability and virulence, although the small sample volume compromises its strength. By incorporating previously experimental studies, we collected several candidates that may support this linkage, which may worth further experimental exploration. For example, urease, an enzyme that catalyzes the hydrolysis of urea into carbon dioxide and ammonia, has been reported to enhance survival of pathogens that experience acidic environments, such as *Yersinia pseudotuberculosis* (Hu et al., 2009), *Helicobacter pylori* (Stingl et al., 2002; Schoep et al., 2010), *Haemophilus influenzae* (Murphy and Brauer, 2011), and *Edwardsiella ictaluri* (Booth et al., 2009), by neutralization of the acidic environments. Interestingly, urease has also been suggested to be a virulence factor that contributes to the persistence and replication in low pH intracellular compartments of macrophages (Booth et al., 2009; Murphy and Brauer, 2011). Such a linkage shows that bacterial resistance to abiotic stresses may be a contributor to virulence.

Another abiotic stress that bacteria frequently encounter is ROS, such as superoxide anion radical, hydrogen peroxide, and hydroxyl radicals. ROS causes damages to DNA, RNA, protein, and lipids (Cabiscol et al., 2000). For example, intestinal mucosa uses epithelial NADPH oxidases to generate ROS for defending against *Campylobacter jejuni* infection, impairing bacterial capsule formation and decreasing *C. jejuni* virulence (Corcionivoschi et al., 2012). Oxidative stress has also been reported to affect the pathogenicity of *E. coli* by decreasing the expression of all virulence factors (Hegde et al., 2008). In addition, when invading hosts, bacterial pathogens risk being phagocytosed by phagocytes that generate superoxide and other ROS in order to kill these intruders (Hassett and Cohen, 1989). Environmental factors, such as desiccation and near UV radiation, can also generate intracellular oxygen ions (Cabiscol et al., 2000). In order to survive these harsh conditions, bacteria develop a set of mechanisms for sensing ROS acutely and maintaining ROS at a certain steady level. Once bacteria sense high-level ROS, defense mechanisms are initiated for getting rid of ROS by expressing antioxidant enzymes, such as superoxide dismutase and catalase, and repairing oxidative damages, such as initiating DNA-repair systems.

Take *B. pseudomallei* and *Salmonella typhimurium* for example. Both of the bacteria are human pathogens that are capable of intracellular survival in human macrophages and can synthesize superoxide dismutase (SOD, EC = 1.15.1.1), an enzyme that changes superoxide, a compound with superoxide anion (O_2_^-^) generated by passing a single electron to molecular oxygen (Shvinka et al., 1979), into oxygen and hydrogen peroxide (H_2_O_2_). Comparisons of wild type and SOD-deficient strains of both pathogens revealed that viabilities of SOD-deficient strains decrease and presence of SOD is required for pathogen virulence (De Groote et al., 1997; Vanaporn et al., 2011). However, hydrogen peroxide is still a comparatively strong oxidizer for bacteria. Thus, in order to avoid its damage, many bacteria possess an enzyme known as catalase, which can decompose H_2_O_2_ into H_2_O and O_2_. Mandell (1975) studied catalase functions in *Staphylococcus aureus* and concluded that catalase has a role in protecting intraphagocytic microbes and may be an important virulence factor. Recent investigation confirmed the potential role that catalase plays for the virulence of pathogens by studying *Leptospira interrogans* (Eshghi et al., 2012). However, this is not a solid relationship. A study of *Salmonella typhimurium* showed that catalase mutant was resistant to macrophages and retained full virulence (Buchmeier et al., 1995). Another study focusing on *Staphylococcus aureus* showed that catalase-negative strain retained virulence in a mouse model of chronic granulomatous disease, which contradicted with the result reported previously (Messina et al., 2002). Thus, although antioxidant enzymes are widely accepted as bacterial protectors against oxidative stresses, their role as a virulence factors should be investigated more deeply.

Desiccation is also a common abiotic stress for free-living stage bacteria in drought-like conditions. For example, many nosocomial pathogens have the ability to persist on dry inanimate surfaces, such as scissors, metal plates, and fabrics etc., in order to increase their opportunities to transfer from one patient to another, which is why inanimate surfaces are often considered as the source for outbreaks of nosocomial infections (Kramer et al., 2006). However, bacterial desiccation resistance varies. *Helicobacter pylori*, a Gram-negative bacterium causing non-lethal gastritis, can only survive for less than 90 min on dry surfaces while *Corynebacterium diphtheriae*, a pathogenic bacterium that causes diphtheria with fatality rates of 36.5%, is able to persist on dry surfaces for up to 6 months (Kramer et al., 2006). This empirical evidence also indicates a possible linkage between ASR and virulence. Currently, several desiccation resistance mechanisms have been reported for bacteria. The most well-known mechanism is to use non-reducing disaccharides, trehalose and sucrose, as desiccation stress protectants. Trehalose is a disaccharide with a α-1,1-glucosidic bond linking two glucosyl residues. Sucrose is composed of glucose and fructose. Non-reducing disaccharides increase bacterial desiccation tolerance mainly by lowering the temperature of dry membrane phase transition and maintaining general protein structure (Leslie et al., 1995). In addition, these disaccharides are responsible for the formation of vitreous cytoplasmic matrix, a virulence factor for bacterial pathogenicity (Garcia, 2011). Besides, trehalose metabolism is also interlinked with glycogen and maltose pathway, the later contributing to *E. coli* O157:H7 colonization in the intestine of mice hosts (Jones et al., 2008). The relationship between trehalose, maltose, and glycogen could serve a good example for tackling the sit-and-wait hypothesis experimentally by linking ER, ASR, and virulence all together.

## Conclusion

In this study, we systematically explored the recent progress of the sit-and-wait hypothesis. Epidemiological data were collected to investigate the relationship between durability and virulence through representative bacterial pathogens. Our analysis provides preliminary evidence that non-vector-borne pathogens with sit-and-wait potentials investigated in this study have higher durability and virulence compared with other groups of bacteria, although the conclusion is subjected to limited bacterial sample volume. In addition, two sets of HMMs were used to scan proteomes from a sample set of bacterial species for proteins related ASR and the presence of durable energy storage pathways, while the presence of virulence factors was assessed via sequence searches. Differential patterns were identified in terms of bacterial proteome size, ESMs, ASR, and also virulence factors. All of these results support that sit-and-wait pathogens have multiple ESMs and higher number of durability and virulence genes. It is noteworthy that our results are based on a small sample volume, that is, only five species for each category. However, our study is the first theoretical support for the hypothesis at the genetic level through bioinformatics methodologies. We urge that further efforts should consider exploring the topic with an increased sample size through similar methods. In addition, this study also provides protein targets for experimentally investigating the hypothesis, such as proteins related with ESMs and also ASR. In conclusion, molecular experimental studies should be performed in order to provide evidence at the cellular and population levels for this hypothesis.

## Author Contributions

LW and MW conceived the core idea and wrote the manuscript. MW also contributed to the statistical analysis. LW and ZL did all data collection and statistical analysis. SD and JY contributed to data analysis and manuscript writing.

## Conflict of Interest Statement

The authors declare that the research was conducted in the absence of any commercial or financial relationships that could be construed as a potential conflict of interest.
