# Supplementary material for: The Sit-and-Wait Hypothesis in Bacterial Pathogens: A Theoretical Study of Durability and Virulence
Source: Front Microbiol. 2017 Nov 3;8:2167. doi: 10.3389/fmicb.2017.02167 (PMC5701638; doi:10.3389/fmicb.2017.02167)
Supplement: Supplementary file 1 [file Table_1.DOCX]

**Supplementary Table S1** GO identifiers used for collecting seed proteins related with bacterial abiotic stress resistance.

| **GO Term** | **GO Annotation** |
| --- | --- |
| GO:0034605 | Cellular response to heat |
| GO:0010447 | Response to acidity |
| GO:0071474 | Cellular hyperosmotic response |
| GO:0009411 | Response to UV |
| GO:0009269 | Response to desiccation |
| GO:0001666 | Response to hypoxia |
| GO:0009409 | Response to cold |
| GO:0006970 | Response to osmotic stress |
| GO:0009266 | Response to temperature stimulus |
| GO:0009415 | Response to water stimulus |
| GO:0009268 | Response to pH |
| GO:0071456 | Cellular response to hypoxia |
| GO:0070417 | Cellular response to cold |
| GO:0071480 | Cellular response to gamma radiation |
| GO:0071468 | Cellular response to acidity |
| GO:0071465 | Cellular response to desiccation |
| GO:0071470 | Cellular response to osmotic stress |
| GO:0071454 | Cellular response to anoxia |
| GO:0009651 | Response to salt stress |
| GO:0009650 | UV protection |
| GO:0071472 | Cellular response to salt stress |
| GO:0071469 | Cellular response to alkalinity |
| GO:0071453 | Cellular response to oxygen levels |
| GO:0070415 | Trehalose metabolism in response to cold stress |
| GO:0006972 | Hyperosmotic response |
| GO:0071502 | Cellular response to temperature stimulus |
| GO:0070483 | Detection of hypoxia |
| GO:0070482 | Response to oxygen levels |
| GO:0050826 | Response to freezing |
| GO:0042538 | Hyperosmotic salinity response |
| GO:0034644 | Cellular response to UV |
| GO:0034059 | Response to anoxia |
| GO:0016048 | Detection of temperature stimulus |
| GO:0010332 | Response to gamma radiation |
| GO:0009314 | Response to radiation |
