# Supplementary material for: The Sit-and-Wait Hypothesis in Bacterial Pathogens: A Theoretical Study of Durability and Virulence
Source: Front Microbiol. 2017 Nov 3;8:2167. doi: 10.3389/fmicb.2017.02167 (PMC5701638; doi:10.3389/fmicb.2017.02167)
Supplement: Supplementary file 2 [file Table_2.DOCX]

**Supplementary Table S2** Bacterial classification based on distribution patterns of durability and virulence genes. Four groups are defined. For each category, only five representative bacterial species are displayed. ^*^Energy Reserve includes G: Glucose, PP: Polyphosphate, T: Triacylglycerol, W: Wax Ester, and PB: Polyhydroxybutyrates. SUM represents total number of energy storage compound types (0-5). ^#^ASR genes are related to Starvation, Temperature, Osmolarity, pH, and Desiccation, etc. ^##^VF means bacterial virulence factors.

| **Group I Sit-and-wait Pathogens** | | | | | | | | | | | |
| --- | --- | --- | --- | --- | --- | --- | --- | --- | --- | --- | --- |
| **Species** | **Strain** | **PN** | **Energy Reserve^*^** | | | | | | **ASR**^#^ | | **VF^##^** |
|  |  |  | G | PP | T | W | PB | SUM | % | | % |
| *Acinetobacter baumannii* | AB0057 | 3732 | 0 | 1 | 0 | 1 | 0 | 2 | 5.17 | | 4.18 |
| *Burkholderia pseudomallei* | 1710b | 6329 | 0 | 1 | 0 | 0 | 1 | 2 | 4.57 | | 8.11 |
| *Corynebacterium diphtheriae* | NCTC13129 | 2264 | 1 | 1 | 0 | 0 | 0 | 2 | 3.89 | | 3.80 |
| *Mycobacterium tuberculosis* | H37Ra | 3990 | 1 | 1 | 0 | 1 | 0 | 3 | 6.87 | | 4.89 |
| *Yersinia pestis* | D182038 | 3632 | 1 | 1 | 0 | 0 | 0 | 2 | 5.20 | | 11.4 |
| **Group II Vector-borne Pathogens** | | | | | | | | | | | |
| **Species** | **Strain** | **PN** | **Energy Reserve** | | | | | | **ASR** | **VF** | |
|  |  |  | G | PP | T | W | PB | SUM | % | % | |
| *Anaplasma phagocytophilum* | HZ | 1244 | 0 | 0 | 0 | 0 | 0 | 0 | 2.65 | 1.69 | |
| *Borrelia burgdorferi* | JD1 | 1358 | 0 | 0 | 0 | 0 | 0 | 0 | 2.87 | 1.99 | |
| *Ehrlichia chaffeensis* | Arkansas | 1100 | 0 | 0 | 0 | 0 | 0 | 0 | 3.09 | 1.82 | |
| *Francisella tularensis* | FSC147 | 1388 | 1 | 0 | 0 | 0 | 0 | 1 | 4.11 | 5.12 | |
| *Rickettsia rickettsii* | Iowa | 1384 | 0 | 0 | 0 | 0 | 0 | 0 | 3.40 | 2.89 | |
| **Group III Exclusively Host-associated Bacteria** | | | | | | | | | | | |
| **Species** | **Strain** | **PN** | **Energy Reserve** | | | | | | **ASR** | **VF** | |
|  |  |  | G | PP | T | W | PB | SUM | % | % | |
| *Helicobacter pylori* | J99 | 1488 | 0 | 1 | 0 | 0 | 0 | 1 | 3.36 | 11.2 | |
| *Mycoplasma pneumoniae* | ATCC15531 | 629 | 0 | 0 | 0 | 0 | 0 | 0 | 3.02 | 3.82 | |
| *Mycoplasma genitalium* | NCTC10195 | 483 | 0 | 0 | 0 | 0 | 0 | 0 | 4.14 | 6.42 | |
| *Treponema pallidum* | SS14 | 1028 | 0 | 0 | 0 | 0 | 0 | 0 | 3.79 | 3.79 | |
| *Ureaplasma urealyticum* | ATCC33699 | 646 | 0 | 0 | 0 | 0 | 0 | 0 | 2.63 | 1.86 | |
| **Group IV Free-living Bacteria** | | | | | | | | | | | |
| **Species** | **Strain** | **PN** | **Energy Reserve** | | | | | | **ASR** | **VF** | |
|  |  |  | G | PP | T | W | PB | SUM | % | % | |
| *Acidobacterium capsulatum* | DSM11244 | 3363 | 1 | 1 | 0 | 0 | 0 | 2 | 3.30 | 3.72 | |
| *Chloroflexus aurantiacus* | DSM635 | 3850 | 1 | 1 | 0 | 0 | 0 | 2 | 4.34 | 3.51 | |
| *Deinococcus radiodurans* | ATCC13939 | 3085 | 1 | 1 | 0 | 0 | 0 | 2 | 3.01 | 2.27 | |
| *Rhodobacter sphaeroides* | DSM158 | 4285 | 1 | 1 | 0 | 0 | 1 | 3 | 4.15 | 4.57 | |
| *Sphingopyxis alaskensis* | DSM13593 | 3154 | 0 | 1 | 0 | 1 | 1 | 3 | 3.90 | 3.58 | |
